# Association between Arthritis and Migraine: A US Nationally Representative Study Including 2649 Adults

**DOI:** 10.3390/jcm10020342

**Published:** 2021-01-18

**Authors:** Louis Jacob, Jae Il Shin, Guillermo F. López-Sánchez, Josep Maria Haro, Ai Koyanagi, Jong Yeob Kim, Jae Han Kim, Hans Oh, Lee Smith

**Affiliations:** 1 Research and Development Unit, Parc Sanitari Sant Joan de Déu, CIBERSAM, Universitat de Barcelona, 08830 Barcelona, Spain; jmharo@pssjd.org (J.M.H.); a.koyanagi@pssjd.org (A.K.); 2 Faculty of Medicine, University of Versailles Saint-Quentin-en-Yvelines, 78180 Montigny-le-Bretonneux, France; 3 Department of Pediatrics, Yonsei University College of Medicine, Seoul 03722, Korea; SHINJI@yuhs.ac; 4 Vision and Eye Research Institute, School of Medicine, Faculty of Health, Education, Medicine and Social Care, Anglia Ruskin University-Cambridge Campus, Cambridge CB1 1PT, UK; Guillermo.Lopez-Sanchez@aru.ac.uk; 5 Institució Catalana de Recerca i Estudis Avançats (ICREA), Pg. Lluis Companys 23, 08010 Barcelona, Spain; 6 College of Medicine, Yonsei University, Seoul 03722, Korea; crossing96@yonsei.ac.kr (J.Y.K.); jaehan0605@yonsei.ac.kr (J.H.K.); 7 Suzanne Dworak Peck School of Social Work, University of Southern California, 1149 South Hill Street Suite 1422, Los Angeles, CA 90015, USA; hansoh@usc.edu; 8 The Cambridge Centre for Sport and Exercise Sciences, Anglia Ruskin University, Cambridge CB1 1PT, UK; lee.smith@anglia.ac.uk

**Keywords:** arthritis, migraine, cross-sectional study, United States

## Abstract

This study aimed to investigate the cross-sectional association between arthritis and migraine in a large representative sample of the US adult population. The study used data from adults who participated in the RAND American Life Panel (ALP). Arthritis (excluding rheumatoid arthritis) and migraine were self-reported. Control variables included sex, age, ethnicity, marital status, education, employment, annual family income, stroke, epilepsy, coronary artery disease, asthma, depression, anxiety, bipolar disorder, and alcohol dependence. The association between arthritis and migraine was investigated using multivariable logistic regression models, while sex and age interaction analyses were also conducted. This study included 2649 adults (51.7% women; mean (SD) age 50.6 (15.9 years). The prevalence of migraine was 10.7% in the sample. After adjusting for several potential confounders, there was a significant association between arthritis and migraine (OR = 1.83, 95% CI = 1.20–2.81). Further sensitivity analyses revealed that the association was significant in women, adults aged ≤45 years, and those aged >65 years. The mere fact that arthritis and migraine may coexist is problematic, as this could lead to an important medical and economic burden. Therefore, strategies should be implemented to reduce the cooccurrence of these two chronic conditions.

## 1. Introduction

Osteoarthritis is a degenerative joint and age-related disorder affecting the articular cartilage. The most frequently affected joints are the knees, hips, fingers, and the lumbar spine [1]. The worldwide prevalence rates of symptomatic osteoarthritis are 9.6% and 18.0% in men and women aged >60 years, respectively [1]. Other less common forms of arthritis are rheumatoid arthritis, gout, and lupus [2]. More than 54 million people are affected by arthritis in the United States, which accounts for 23% of the adult population [2]. Arthritis is one of the major causes of work disability in this country, with the related cost reaching $303 billion per year [2].

Each arthritis type exhibits unique symptoms, however common symptoms include joint pain, joint inflammation, limitation of joint mobility, warm and red skin over the effected joint, and muscle weakness [3]. Importantly, arthritis-attributable activity limitations affect 45.4% of older adults suffering from arthritis, and around 25% of these adults also report severe pain [4]. These arthritis-related impairments and limitations have been found to negatively impact mental health and health-related quality of life [4].

Another health outcome that arthritis may be associated with is migraine. Migraine is characterized by a moderate or severe headache lateralized to one side of the head, which can also involve nausea, vomiting, and photo- or phonophobia [5]. It has been reported that migraine can lead to substantial levels of disability, while it coexists with a wide range of other disorders [6]. In a study of 1750 migraine patients, frequent migraine triggers were stress (79.7%), hormones in women (65.1%), not eating (57.3%), weather (53.2%), disturbed sleep (49.8%), perfume or odors (43.7%), cervical pain (38.4%), lights (38.1%), alcohol (37.8%), smoke (35.7%), late bedtime (32.0%), heat (30.3%), food (26.9%), exercise (22.1%), and sexual activity (5.2%) [7]. Interestingly, some of these triggers have also been associated with arthritis. For example, those with arthritis have reported sleep disturbance [8], stress and distress [9], as well as neck pain [10]. Moreover, exercise is a recommended non-pharmacological treatment option for those with arthritis, but as highlighted above, it is also a trigger for migraine [11]. Finally, the prescription of analgesic drugs is frequent in patients with osteoarthritis [12], and the use of these drugs may worsen headaches [13]. However, to date, only few studies have examined the association between arthritis and migraine [14,15].

Therefore, the aim of the present study was to investigate the association between arthritis and migraine in a large representative sample of American adults. The hypothesis of this study was that arthritis is positively and significantly associated with migraine. Given that there are also important sex and age differences in the epidemiology of arthritis and migraine [16,17,18,19], it was further speculated that the relationship between these two chronic disorders would vary between men and women and between younger and older adults.

## 2. Experimental Section

### 2.1. Study Participants

**Figure 1:**
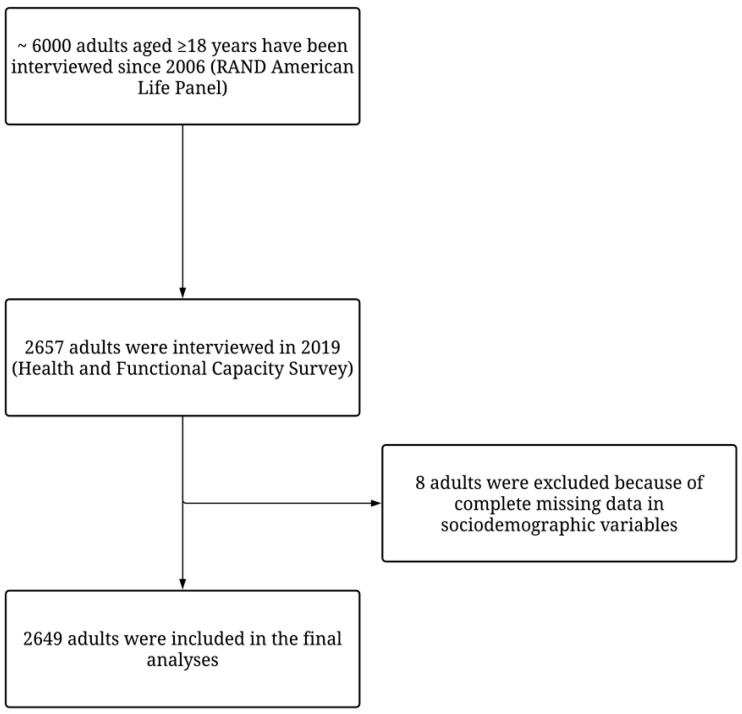
Flow chart of study participants.

This study used data from adults who participated in the RAND American Life Panel (ALP) [20]. The ALP is a US nationally representative panel of more than 6000 individuals aged ≥18 years from around 4500 households who have been interviewed on a regular basis via the Internet since 2006. The flow chart of the study is displayed in Figure 1, with 2649 adults included in the present study. Sampling weights were constructed to account for non-response and the probability of being selected using population distributions from the Current Population Survey (CPS) Annual Social and Economic Supplement administered in March each year [20]. The study sample was representative of the US civilian and residential population aged ≥18 years. Finally, all participants gave their consent, and this research was approved by the RAND’s Human Subjects Protection Committee [21].

### 2.2. Measures

Arthritis (independent variable): In 2019, participants were asked the following two yes/no questions: “Do you suffer from arthritis?” and “Do you suffer from rheumatoid arthritis?”. In order to increase the relative prevalence of osteoarthritis among participants with arthritis, individuals reporting rheumatoid arthritis were excluded from the arthritis group. Previous research has showed that self-reported arthritis is validated in the general population [22].

Migraine (dependent variable): Given that self-reported migraine is highly and positively correlated with clinical migraine [23], in 2019, migraine was assessed with the question: “Do you suffer from migraine?” (yes and no answers).

Control variables: The present study included sex (male and female), age (in years), ethnicity (White/Caucasian and other), marital status (single/separated/divorced/widowed and married/in a domestic partnership), education (≤primary/secondary and ≥tertiary), employment (yes and no), annual family income (<$20,000, $20,000–<$40,000, $40,000–<$60,000, $60,000–<$75,000, and ≥$75,000), stroke (yes and no), epilepsy (yes and no), coronary artery disease (yes and no), asthma (yes and no), depression (yes and no), anxiety (yes and no), bipolar disorder (yes and no), and alcohol dependence (yes and no). All control variables were assessed in 2019, except employment, which was assessed in 2017 and 2018. Furthermore, there were missing data in other sociodemographic variables for around 10.5% of the sample, and these missing observations were filled in using data obtained in 2017 and 2018.

### 2.3. Statistical Analyses

Differences in the sample characteristics by arthritis and migraine status were assessed by chi-squared tests for all variables except age (*t*-tests). The prevalence of migraine was also studied in the overall population and in sex and age subgroups. The association between arthritis (independent variable) and migraine (dependent variable) was further analyzed in the overall population using a logistic regression model adjusted for several potential confounders (sex, age, ethnicity, marital status, education, employment, annual family income, stroke, epilepsy, coronary artery disease, asthma, depression, anxiety, bipolar disorder, and alcohol dependence). After conducting interaction analyses by including the product terms of arthritis X sex and arthritis X age in the regression model, the relationship between arthritis and migraine was also investigated in sex- and age-stratified samples (men, women, adults aged ≤45 years, those aged 46–65 years, and those aged >65 years). The results from the logistic regression analyses are presented as odds ratios (ORs) and 95% confidence intervals (CIs). There were missing data only for employment (0.4%; N = 10) and annual family income (0.2%; N = 4), and a complete-case analysis was carried out. The sample weighting was taken into account in all analyses. The *p*-values < 0.05 were considered statistically significant. All analyses were performed with R 3.6.2 (The R Foundation) [24].

## 3. Results

**Figure 2:**
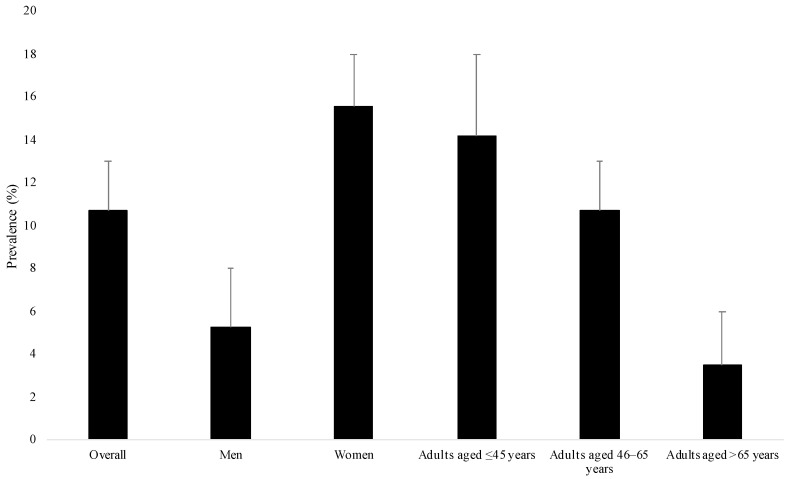
Prevalence of migraine in the overall population and in sex and age subgroups.

**Figure 3:**
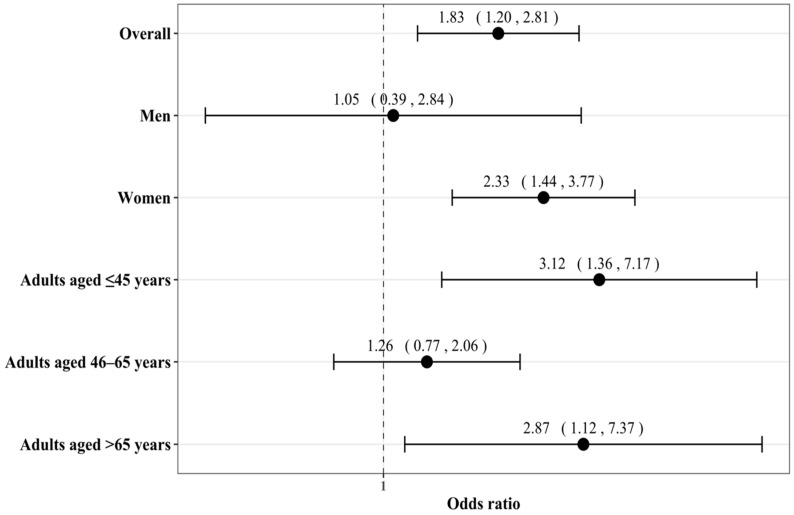
Association between arthritis and migraine in adults living in the US.

This study included 2649 adults (51.7% women; mean (standard deviation) age 50.6 (15.9) years; Table 1). There were 796 participants with and 1853 participants without arthritis. Female sex, older age, no employment, coronary artery disease, asthma, depression, and anxiety were more frequent in people with arthritis. The prevalence of migraine was 10.7% in the overall population, and this prevalence was significantly higher in women and younger adults than in men and older adults, respectively (Figure 2). The results of the regression analyses are displayed in Figure 3. After adjusting for several potential confounders (sex, age, ethnicity, marital status, education, employment, annual family income, stroke, epilepsy, coronary artery disease, asthma, depression, anxiety, bipolar disorder, and alcohol dependence), there was a positive and significant association between arthritis and migraine (OR = 1.83, 95% CI = 1.20–2.81). Interaction analyses showed that sex tended to be a significant effect modifier in the arthritis–migraine relationship (*p*-value = 0.058), and the association was only significant in women (OR = 2.33, 95% CI = 1.44–3.77). Age further modified the association between arthritis and migraine, which was found to be significant in adults aged ≤45 years (OR = 3.12, 95% CI = 1.36–7.17) and aged >65 years (OR = 2.87, 95% CI = 1.12–7.37).

Migraine was assessed with a yes/no question. The prevalence of migraine was compared between the different sex (i.e., male and female) and age subgroups (i.e., age ≤45, 46–65, and >65 years) using chi-squared tests. The *p*-values were lower than 0.001.

Arthritis and migraine were assessed with yes/no questions. The association between arthritis and migraine was studied in the overall population and in sex- and age-stratified samples. Logistic regression models were adjusted for sex (except the sex-stratified analyses), age, ethnicity, marital status, education, employment, annual family income, stroke, epilepsy, coronary artery disease, asthma, depression, anxiety, bipolar disorder, and alcohol dependence. All variables except age were included in the regression models as categorical variables. The sample weighting was taken into account in all regression analyses.

## 4. Discussion

In this large representative sample of US adults, the present study found a positive and significant arthritis–migraine relationship in the overall sample, in women, in adults aged ≤45 years, and in those aged >65 years.

This study does support previous studies that have demonstrated associations between arthritis and poor health outcomes [4,14,15]. The identified positive association between arthritis and migraines may be explained by several plausible pathways. First, as previously mentioned, some consequences of arthritis are also triggers for migraines, including sleep disturbances, stress and distress, and neck pain [7,8,9,10]. For example, a study of 429 older adults with knee osteoarthritis from the United States revealed that the prevalence rates of weekly problems with sleep onset, sleep maintenance, and early morning awakenings were 31%, 81%, and 51%, respectively [25]. A cohort study including 133,262 individuals from South Korea further showed that sleep disorders were significantly associated with the incidence of migraine (hazard ratio =1.59) [26]. Moreover, it is important to note the interrelation between pain distress and sleep disturbances. Indeed, pain is a cause of sleep disturbance and sleep disturbance likely plays a key role in pain expression including distress [27]. Second, exercise is recommended as a non-pharmacological treatment for arthritis [11], but has also been shown to be a trigger for migraines [7]. Indeed, physical activity (e.g., exercise, exertion, and straining) was reported as a migraine-precipitating factor by 25% of patients with migraine in a systematic review of 25 studies [28]. Third, inflammation has been implicated in the development of both arthritis [29] and migraines [30]. For example, inflammation of the synovium and activation of the inflammatory complement system are major underlying mechanisms of the pathogenesis of knee osteoarthritis, while arteriolar vasodilation and plasma protein extravasation are likely to be common phenomena during migraine attacks. Given that melatonin has anti-inflammatory properties [31], osteoarthritis-related sleep problems may further aggravate inflammation via dysregulated plasma levels of melatonin [32]. Fourth, gut microbiota and mast cells may also be involved in the relationship between arthritis and migraine [33,34,35]. A significant association between the abundance of *Streptococcus* species in stool microbiome and osteoarthritis-related knee pain was observed in a secondary analysis of the Rotterdam study (N = 1427 participants) [36], while another study(N = 108 individuals) identified a reduced gut microbial diversity in elderly women with migraine compared with matched healthy controls [37], suggesting that gut microbial dysbiosis may be a common denominator between osteoarthritis and migraine.

It should be noted here that the association between arthritis and migraines was found to be significant in women but not in men, with the interaction analysis tending to statistical significance. Indeed, the prevalence of migraine is three times higher in women (18%) than in men (6%) in the US general population, with this figure reaching 43% in women of reproductive age [38]. This high level of migraine in women has been linked to drops in estrogen levels [39]. Importantly, in women, low levels of estrogen are also associated with higher levels of arthritis [40]. In terms of age, the arthritis–migraine relationship was significant in adults aged ≤45 years and in those aged >65 years, but not in those aged 46–65 years. The fact that the association between arthritis and migraine was particularly strong in the elderly may be explained by a tendency towards higher pain sensitivity in old age [41], and pain could trigger migraines more frequently in adults aged >65 years than in their counterparts aged 46–65 years. On contrast, copying may be lower and stress higher in adults aged ≤45 years than in those aged 46–65 years, and it may account for a share of the differential relationship between arthritis and migraine that was observed between the two age groups.

This is one of the first studies to investigate the relationship between arthritis and migraine. Moreover, the large representative sample of US adults is a clear strength of the present study. However, findings from the present study must be interpreted in light of its limitations. First, this is a cross-sectional analysis, so the direction of the association cannot be inferred. Although migraine has been previously identified as a risk factor for certain types of arthritis (e.g., rheumatoid arthritis) [15], it seems unlikely that migraines would be a cause of osteoarthritis. Second, the present study did not collect data on the different types of arthritis. In spite of the fact that rheumatoid arthritis was excluded from the analyses, arthritis may have included conditions other than osteoarthritis (e.g., ankylosing spondylitis and gout), and it is possible that migraines would be more likely in certain types of arthritis than others. Future research should seek to investigate the association between a certain arthritis type and migraine. Third, both the exposure and outcome variables were self-reported, meaning bias cannot be ruled out. One major bias is migraine misdiagnosis, and a substantial proportion of people reporting migraine may have been affected by other pain disorders such as tension type and medication overuse headaches. Fourth, given that these were lifetime variables, it is possible that arthritis and migraine did not occur simultaneously. Fifth, there were no data on behavioral factors (e.g., smoking status, physical activity, and diet), which may have impacted the study results.

## 5. Conclusions

In conclusion, a positive association between arthritis and migraines was suggested in this nationally representative US study. The mere fact that arthritis and migraine may coexist is problematic, as this could lead to an important medical and economic burden. Therefore, strategies should be implemented to reduce the cooccurrence of these two chronic conditions.

## Figures and Tables

**Table 1:** Sample characteristics (overall and by arthritis and migraine status).

Characteristics	Category	Overall (N = 2649)	Arthritis		*p*-Value ^1^	Migraine		*p*-Value ^1^
			No (N = 1853)	Yes (N = 796)		No (N = 2377)	Yes (N = 272)	
Sex	Male	48.3	51.2	39.2	0.001	51.2	24.2	<0.001
	Female	51.7	48.8	60.8		48.8	75.8	
Age (years)	Mean (standard deviation)	50.6 (15.9)	47.3 (15.1)	60.9 (13.9)	<0.001	51.3 (16.1)	44.7 (13.1)	<0.001
Ethnicity	White/Caucasian	72.9	71.1	78.8	0.054	72.8	73.9	0.792
	Other	27.1	28.9	21.2		27.2	26.1	
Marital status	Single/separated/divorced/widowed	41.0	40.0	43.9	0.278	40.6	44.3	0.446
	Married/in a domestic partnership	59.0	60.0	56.1		59.4	55.7	
Education	≤Primary/secondary	38.4	36.8	43.2	0.098	39.3	30.3	0.096
	≥Tertiary	61.6	63.2	56.8		60.7	69.7	
Employment	No	42.0	35.9	61.4	<0.001	42.2	40.2	0.688
	Yes	58.0	64.1	38.6		57.8	59.8	
Annual family income	<$20,000	14.1	14.4	13.0	0.538	13.9	15.2	0.909
	$20,000–<$40,000	19.2	18.3	21.9		19.5	16.2	
	$40,000–<$60,000	16.4	15.8	18.1		16.2	18.1	
	$60,000–<$75,000	13.4	13.5	12.8		13.4	13.2	
	≥$75,000	37.0	37.9	34.2		37.0	37.3	
Stroke	No	99.1	99.2	98.6	0.110	99.2	98.2	0.093
	Yes	0.9	0.8	1.4		0.8	1.8	
Epilepsy	No	99.5	99.5	99.8	0.269	99.7	98.3	0.029
	Yes	0.5	0.5	0.2		0.3	1.7	
Coronary artery disease	No	98.3	98.9	96.4	<0.001	98.1	99.6	0.003
	Yes	1.7	1.1	3.6		1.9	0.4	
Asthma	No	90.6	91.7	87.0	0.018	92.1	78.5	<0.001
	Yes	9.4	8.3	13.0		7.9	21.5	
Depression	No	81.5	83.2	75.9	0.003	84.8	53.9	<0.001
	Yes	18.5	16.8	24.1		15.2	46.1	
Anxiety	No	79.0	81.1	72.6	0.011	82.1	53.2	<0.001
	Yes	21.0	18.9	27.4		17.9	46.8	
Bipolar disorder	No	97.8	98.1	96.6	0.101	98.2	93.9	0.008
	Yes	2.2	1.9	3.4		1.8	6.1	
Alcohol dependence	No	98.5	98.5	98.4	0.967	98.4	98.9	0.588
	Yes	1.5	1.5	1.6		1.6	1.1	

Arthritis and migraine were assessed with yes/no questions. Data are percentages unless otherwise stated. The sample weighting was taken into account in the analyses; ^1^ *p*-values were based on chi-squared tests except for age (*t*-tests).

## Data Availability

RAND ALP datasets are available after registration at https://alpdata.rand.org/.
